# Retraction: MicroRNA-542-3p inhibits oral squamous cell carcinoma progression by inhibiting ILK/TGF-β1/Smad2/3 signaling

**DOI:** 10.18632/oncotarget.28798

**Published:** 2025-12-31

**Authors:** Bin Qiao, Jing-Hua Cai, Alfred King-Yin Lam, Bao-Xia He

**Affiliations:** ^1^Department of Stomatology, The First Affiliated Hospital of Zhengzhou University, Zhengzhou 450052, P.R. China; ^2^Cancer Molecular Pathology, School of Medicine and Menzies Health Institute Queensland, Griffith University, Gold Coast 4222, Australia; ^3^Department of Pharmacy, Affiliated Cancer Hospital of Zhengzhou University/Henan Cancer Hospital, Zhengzhou 450003, P.R. China; ^*^These authors shared the co-first author

**This article has been retracted:** An Oncotarget Image Forensics investigation has discovered instances of internal and external image overlaps and duplications. The GAPDH bands in Figure 1B also appeared as b-actin blot in Figure 6B. This b-actin blot was found in the papers published concurrently in Figure 3A of [1], Figure 4B of [2] and in Figure 2C of [3], This blot also appeared as GAPDH blot in papers [4–6]. All these papers have been retracted since. The TGF-b1 blot in Figure 1B was found as Ngfap1 blot in later published paper [7]. Figure 9A, representing transwell assay data, contains internal overlaps as well as overlaps with images in later published papers [8–10]. Additionally, we found duplicated tumor images in Figure 11B, some of which also appeared in already retracted papers [11, 12]. The authors were notified of the problems and the corresponding author Dr. Bin Qiao has claimed the images are original and that they are the victim of image leaking, but they have not provided suitable documentation to demonstrate originality or an adequate explanation of how the alleged leaking occurred. Given the number and timing of these duplications, the Editors have determined that the integrity of the published work is compromised. Therefore, the decision has been made to retract this paper.

Original article: Oncotarget. 2017; 8:70761–70776. 70761-70776. https://doi.org/10.18632/oncotarget.19986
